# A qualitative evaluation of the crucial attributes of contextual Information necessary in EHR design to support patient-centered medical home care

**DOI:** 10.1186/s12911-015-0150-x

**Published:** 2015-04-16

**Authors:** Charlene R Weir, Nancy Staggers, Bryan Gibson, Kristina Doing-Harris, Robyn Barrus, Robert Dunlea

**Affiliations:** VA HSR&D Informatics and Decision Enhancement Center of Innovation (IDEAS), 500 Foothill Blvd, Salt Lake City, UT 84108 USA; Department of Biomedical Informatics, School of Medicine, University of Utah, Suite 208, 421 Wakara Way, Salt Lake City, 84108 UT USA; School of Nursing, University of Maryland, 522 West Lombard Street, Baltimore, MD 21201 USA; Division of Epidemiology, School of Medicine, University of Utah, 295 Chipeta Way, Salt Lake City, Utah 84132 USA

**Keywords:** Patient medical home, Cognitive task analysis, Contextual information, Electronic health record, Patient preferences, Information access

## Abstract

**Background:**

Effective implementation of a Primary Care Medical Home model of care (PCMH) requires integration of patients’ contextual information (physical, mental, social and financial status) into an easily retrievable information source for the healthcare team and clinical decision-making.

This project explored clinicians’ perceptions about important attributes of contextual information for clinical decision-making, how contextual information is expressed in CPRS clinical documentation as well as how clinicians in a highly computerized environment manage information flow related to these areas.

**Methods:**

A qualitative design using Cognitive Task Analyses and a modified Critical Incident Technique were used. The study was conducted in a large VA with a fully implemented EHR located in the western United States. Seventeen providers working in a PCMH model of care in Primary Care, Home Based Care and Geriatrics reported on a recent difficult transition requiring contextual information for decision-making. The transcribed interviews were qualitatively analyzed for thematic development related to contextual information using an iterative process and multiple reviewers with ATLAS@ti software.

**Results:**

Six overarching themes emerged as attributes of contextual information: Informativeness, goal language, temporality, source attribution, retrieval effort, and information quality.

**Conclusions:**

These results indicate that specific attributes are needed to in order for contextual information to fully support clinical decision-making in a Medical Home care delivery environment. Improved EHR designs are needed for ease of contextual information access, displaying linkages across time and settings, and explicit linkages to both clinician and patient goals. Implications relevant to providers’ information needs, team functioning and EHR design are discussed.

## Background

The Patient Centered Medical Home (PCMH) model of health-care delivery is becoming a national model of care and a high priority of the Veterans Administration (VA). The goals of PCMH are to partner with patients, increase access to care, improve coordination and enhance team functioning. To accomplish these goals, PCMH teams must integrate the patient’s social information, functional status and personal context into medical decision-making and collaborative care planning. Effective PCMH implementation therefore requires that patients’ contextual information be easily available and in a form that is useful for healthcare providers [1-3]. Very little is known about the requirements for displaying contextual information that would be most effective in supporting clinical decision-making. The objective of this study was to address this gap in order to inform the development of a next-generation EHR that could effectively support the PCMH model of care. In other words, acquiring an understanding of where and how clinicians look for functional information and what form they view as useful will be a first step forward in improving EHR functionality.

### Contextual information

Patient context refers to aspects of patients’ environment, behavior, social, financial or functional status (Table 1). If available and in a usable form, contextual information can provide powerful prognostic and therapeutic tools to the clinician’s armamentarium.

**Table 1:** **Categories of contextual information**

***Physical status***	Information about the physical functioning of the patient, including the ability to physically manage common, everyday tasks for self-care. Sub-elements of the category include, for example, patients’ mobility, walking, balance, strength and coordination, ability to dress and feed themselves.
***Mental status***	Mental status refers to information basic cognitive capabilities for self-care. It includes complex or health-related decision-making; managing finances or life events; the ability to comprehend information such as medication-related information; and/or the cognitive aspects of hygiene and nutrition.
***Social status***	This category includes information about patients’ functioning within the social roles, e.g. marital status, participation in activities outside the home and social support. Includes involvement in organizations, participation in church, or attendance at social events outside the home.
***Financial status***	This category addresses patients’ fiscal capabilities to provide for themselves, especially for basic life necessities (food, housing) and healthcare needs (medications, ability to afford home care or nursing home).

### Contextual errors and decision-making

Contextual error is defined as “decision-making errors that occur because of inattention to patient context [4-7]”. Weiner et al. developed a provocative theory of medical information by dividing clinical decision-making into two distinct cognitive skills: 1) biomedical decision making - the ability to classify patient’s conditions into diagnostic and management categories; and 2) contextual decision making - the skills to apply social, financial, mental and physical function information to the patient’s individual condition [6,8]”.

When clinicians do not use contextual information in care planning, the result is poorer quality of care [5]. Contextual errors represent a failure to individualize evidence based care to patient’s unique situation. For example, knowledge about increasing levels of glucose requires clinicians to understand the pathophysiology of diabetes. Discovering that the patient’s caretaker is ill and linking that to the increasing blood glucose levels requires clinicians to elicit and integrate contextual information about the patient’s functional status and dietary habits.

Clinicians often underutilize contextual information [9-14]. Studies reveal that functional status information is often not assessed and if it is assessed, functional status is often not recorded in the electronic health record [15-17]. For example, emergency room physicians rarely assessed functional status and were found to incorrectly diagnose a disability almost half of the time. If they had considered just two activities of daily living in their assessments, their clinical judgment of disability would have improved significantly [17]. More often, the failure is not one of collecting the information but rather of retrieving contextual information collected by other members of the team in a variety of different forms [18]. In our experience, nurses and social workers are the most likely to collect contextual information [19], but their notes are rarely read by physicians [20].

### The veteran’s health administration’s computerized patient record system (CPRS)

The U.S. Veterans Health Administration (VHA) provides care to approximately 9 million veterans using an EHR known as Computerized Patient Record System (CPRS). CPRS is structured analogously to a paper chart with much of the information stored as free text. VA clinicians have been using CPRS electronic notes since 2001-2002; therefore, their documentation and information retrieval patterns are well established. However, finding pertinent information in CPRS can be challenging. For example, a recent review found that 33,000 surgical patients each had on average 400 free text documents with a total of 1.5-2.0 megabytes each of free-text data [21]. Thus, this study was designed to explore the perceptions of clinicians on the availability and attributes of contextual information needed for clinical decision-making.

## Methods

### Design

This qualitative study used cognitive task analysis (CTA) [22] techniques. CTA addresses the perceptions, goals, beliefs and thought processes of individuals while they engage in a task. We integrated CTA with the Critical Incident Technique, a method to investigate participants’ goals and thoughts during a particular event they selected as being important and illustrative to a defined issue, adapting Crandall, Klein’s and Hoffman’s format to the clinical situation [22,23]. We have found that when individuals are recalling specific events, they are much more informative (although not totally without bias) than when discussing what they “usually do”. We confirm our adherence to the Qualitative Research Review Guidelines (RATS) recommended by the BioMed Central journals.

### Setting and participants

The study was conducted at the Salt Lake City VA Medical Center. The University of Utah IRB approved the study, as did the local VA research office. To reflect the typical composition of PCMH teams, participants were recruited from three departments 1) Home Based Primary Care (HBPC) (n = 4); 2) Geriatric Home-based discharge program (G-Help) (n = 2), and 3) SLC VAMC Medical Home based primary care clinics (n = 11). Provider roles included 7 Social Workers, 6 Nurse Practitioners, 2 resident MDs, 1 attending MD, and 1 nurse Case Manager. Recruitment was face-to-face and in staff meetings (after getting permission from the coordinator of the PACT team). The proportion of clinicians reflect the membership on the VAs medical home team called Patient Aligned Care Teams (PACT) Seventeen of the 20 possible providers completed the study (85% response rate). All participants were currently members of a PACT team. Participants had worked in the VA for an average of 11 years. Participants mean self-rated CPRS skill level was 3 on a 4-point scale. All participants were consented per IRB requirements (including confirming rules of confidentiality).

### Procedures

After introducing the study to managers and staff, participants were solicited through internal email. The four interviewers (RD, CW, and NS), all clinicians, were trained in using the interview script. Two of the interviewees had both clinical training as an RN and are either trained in user-centered design (NS) or have advanced degrees in Cognitive Psychology (CW). The other interviewer was a physician who had not yet started his medical residency. All three have not been in the clinical setting caring for patients for longer than 3 years – and sometimes as long as 20. The 2 senior researchers have had extensive training in qualitative techniques in order to minimize bias as well as human factors advanced degrees. The script consisted of semi-structured questions with probes used as needed to capture rich data from each participant using a 3-wave process of describing the patient, elaborating on the timeline and then responding to specific probes.

Providers were consented and informed that all identifying information would be removed from the transcript and the data would be stored on the VA server requiring password entry from only our team. Participants were asked to recall a recent patient who was involved in a difficult transition of care (e.g. discharge from the hospital), where contextual information was needed for decision-making. They were asked to open the patient’s chart in CPRS, review their last progress note and retrieve any associated documentation up to the point of their last interaction with the patient (to avoid bias in knowing the eventual disposition of the patient participants did not read notes written after their own last note). The emphasis was on their experience, and every effort was made to avoid creating an environment where the participant would feel they were being judged or evaluated. Actions to minimize evaluation anxiety included the following: 1) we explained that the intent was to improve the computer, not their practice patterns; 2) we emphasized that there would be no right or wrong answers; and 3) we were alert to signs of defensive responses. Interviews lasted approximately 30 minutes and were audio recorded. We interviewed all those who volunteered. The recordings were professionally transcribed and all personal identifiers were removed in the process of transcription.

### Analysis

Qualitative analysis of the transcripts was done using procedures congruent with thematic development [24]. The entire research team participated in the analyses using ATLAS.ti qualitative software and decisions made through consensus. The process was iterative consisting of multiple rounds of reading and discussion using inductive processes. Data analysis followed the steps recommended by Patton: (1) provisional pre-coding to establish definitions and boundaries among constructs, (2) group discussion and consensus coding of all of the transcripts, (3) consolidation of categories after reviewing the associated quotations; and 4) an additional discussion and review of the categories and quotations to identify emergent themes [25]. Once the research team agreed on the general approach, each member of the team separately and independently highlighted all of the transcripts using what Patton refers to as “pre-codes”, where phrases and sections of text are given a pre-code (a short string of words or phrases that is very close to the text) by the reader that is as close as possible to the text. The next round consisted of grouping similar codes together into 80 constructs. These were collapsed into 35 categories by discussing meanings and relationships across various constructs. The final round linked categories into a total of six themes resulting in a taxonomy of contextual information characteristics. A preliminary version of the taxonomy was presented at a conference [26]. Further review and analyses clarified the themes, conceptual boundaries and associated attributes in the taxonomy. Member checking of 3 participants was conducted. Quotes were chosen collectively in terms of their representativeness of each category.

## Results

### Overview

The six themes from the analyses represented attributes of contextual information clinicians deemed important across all four categories of contextual information described earlier in Table 1. Table 2 presents the six overarching themes of contextual information found in our analysis of the transcripts.

**Table 2:** **Attributes of contextual information**

**Attribute**	**Description**
***Informativeness***	Refers to task relevance, usefulness, vividness and clarity
***Goal language***	Abstract language referencing values, clinical goals, patient perspectives and desired objectives Includes language related to cause.
***Temporality***	Information related to attributes of time, including trending, baselines, and future temporal expectations regarding clinical metrics.
***Source attribution***	Information from various sources is valued differently. Source includes clinical role, setting and known responsibilities.
***Retrieval effort***	Extraction of contextual information requires significant effort. A tension exists between titrating the effort required versus perceived importance.
***Information quality***	Refers to beliefs regarding the completeness and accuracy of information, largely determined by redundancy, source quality and trust.

### Informativeness

Informativeness refers to the utility of a piece of information for a specific clinical decision. Two components of informativeness described by respondents as particularly important were relevance and vividness.

Information varies on how relevant it is as a function of the particular decision at hand. For example, knowing whether a patient can’t drive might not be relevant to deciding on hypertension medications but might play an important part in the decision to order warfarin because of the patient’s need for transportation for frequent monitoring (laboratory tests). As a result, the relevance of contextual information is highly idiosyncratic. In one example, a young patient was wheelchair bound and being considered for admission to a drug rehab inpatient unit. Although he was able to care for himself and had lived many years independently, the nurses on the drug rehab unit looked at the fact he was wheelchair-bound and thought he would have extensive care needs so were reluctant to admit him. More than usual relevant contextual information about his living situation and functional status (independent) was required to make the decision for admission. In another example, clinicians had to decide if an elderly patient could stay at home or go to a group living center but could not determine if they had adequate social information. The text below illustrates that a clinician was providing information about the “quality” of the data itself, noting that without family providing information, it is hard to tell how the patient is doing.

*“He’s pretty much alienated himself from most of the family because of his cantankerousness…so he’s pretty isolated.”*

Another component of informativeness is *vividness*. Vivid narrative is compelling and evokes meaningful images. For example, the following text from a patient’s chart is especially vivid, ***“ . . .****the patient crawled to the mailbox every day to get his mail.”* Vivid information often trumps other information by supporting the ability of clinicians to easily form a mental model of patients, their capabilities and their situations.

In both of these cases, the most informative narrative (and by report of the clinicians, the most desirable) was not found in structured formats. Clinicians were able to identify components of notes that were imbedded templates versus open-ended text.

### Goal language

Goal language refers to information regarding the patients’ values, personal preferences, and long-term aspirations. Participants reported that effective clinical goal creation requires knowledge of the patient’s personal goals as well as other contextual information. In the words of one participant:

*“The overall goal of the medical team is to find a solid discharge plan for him where again he won’t be a bounce back because of social issues or because of the high increased needs of health care giving.”*

*“The patient wanted to stay home, so we tried to figure out a way to make it happen as best we could.”*

### Temporality

Participants universally sought information related to the timing (onset, ending, fluctuation patterns) over time for contextual information. Questions, such as “When was that true?” or “How long has that been true?” are essential to achieving a full understanding of the patient. For example, clinicians wanted to understand how functional status changed over time, the rate of change and basic baseline information:

“*He technically doesn’t have full-blown dementia yet, but he’s definitely getting more and more forgetful over the past year.”*

Participants reported that the temporality of information was especially hard to acquire without extensive sorting and skimming through progress notes from a diverse set of authors.

### Source attribution

Knowing the source of the information was as often as important as the content of the information itself. Interviewees appeared to have extensive implicit theories about the value of a source related to the information task at hand, e.g., social worker’s notes were better sources for social information than physician’s notes. Formal assessments of a contextual domain were given more weight than other sources, e.g., an evaluation by an occupational therapist or financial advisor were more valued than an informal comment in the notes by a resident In addition, information conveyed verbally was given increased weight than information via email or notes:

*“Verbally he told me what his goals were. And then I also had background because the doctor who saw him with me knew him for the past four years . . . ”*

Finally, in a specific setting a person with first-hand knowledge of the patient’s functional capacity could be considered a more credible source of information than the patient himself.

*“ . . .. because the daughter-in-law is probably the least emotionally involved, but has the best information. In other words, her husband tends to sort of side with his mother. He sometimes isn’t realistic.”*

Or, when the information comes from a source that has questionable veracity or is unknown, then there is considerable concern about the reliability and integrity of the information provided.

“And that’s the other thing is, I had to totally put my trust in this woman named ***, that I had never met before. I just had to take it as a fact that she was his daughter.”

### Retrieval effort

A key attribute of contextual information is the effort required to retrieve it. Participants repeatedly commented about the difficulty of extracting contextual information because it is “buried” in notes. Effort is required first to locate the information and then determine its relevance, source, temporal attributes and quality. Clinicians seemed to have well-developed mental models of how much effort would be involved in retrieving different forms of information in the EHR:

“*I don’t know where I would gather that other than I would assume a lot of that is in the homeless diagnosis*.”

“*Sorting through the myriad of notes in order to find something about his ability to care for himself is exhausting. I just don’t do it*.”

Participants commented that the amount of effort to retrieve data is weighed against its relevance to the particular decision at hand and balanced against the search time required. Providers stated they would spend more effort searching for data when first assuming care or when the data was deemed absolutely essential. The ratio of effort versus value was mentioned frequently. Otherwise, imperfect data searching was the rule. For complex decisions requiring detailed contextual information, participants reported that in-person multidisciplinary meetings were essential because they were more effective than attempting to retrieve these same data by scouring the EHR. Several participants resorted to calling individuals who recently saw the patient to locate needed information.

### Information quality

Information quality refers to the consistency, completeness and accuracy of the content. Completeness refers to both comprehensiveness of the content and whether enough detail is provided for a specific decision. Clinicians described schema regarding the relationship of the quality of the contextual information to a specific domain. For example, if a patient is dying, information about their preferences, pain needs and social support are needed to determine disposition, e.g., inpatient hospice or a home death. But the information is often inconsistent.

*“ . . . not having initial information and the patient’s ability to give much corroborating history and I will fill in those details. So that we really pressed him for a sense of his functional status and his social network and all of those kind of things. We were not very confident in the veracity of those, of his self-reports in that regard.”*

Information accuracy has two dimensions, the degree of redundancy and the trustworthiness of the source. Providers reported looking for contextual information and purposively used multiple sources to confirm or disconfirm information.

## Discussion

This project explored clinicians’ perceptions about important attributes of contextual information for clinical decision-making; how contextual information is expressed in CPRS clinical documentation as well as how clinicians in a highly computerized environment manage information flow related to these domains. The study offers a unique perspective on the contextual information attributes needed to support clinical decision making in patient-centered medical homes and what attributes contextual information must possess to be clinically useful.

The results of this study indicate that useful contextual information is not easily retrievable even in this highly computerized environment. These attributes would be crucial for any patient summary and for creating a shared understanding of the patient between team members. Bringing this information into the hands of clinicians in a useful form is likely beyond simple ontologies or structured data because it is highly idiosyncratic to both the patient’s specific characteristics as well as their unique situation at hand.

### Implications for EHR design

Including contextual, functional and social data in the EHR is beginning to gain increasingly more attention. As the Institute of Medicine’s report entitled “Capturing Social and Behavioral Domains in the Electronic Health Records suggested, the inclusion of social and behavioral information would increase significantly the precision of data, as the note “Social and behavioral data can describe potentially modifiable conditions that, along with clinical and biological data, can provide more preventive, diagnostic, and therapeutic options for improving individual and population health.” (p. 19) [27].

The six attributes identified in this study are relevant to design improvements for improving current EHRs. The first redesign concerns incorporating goal language into the medical record. Extensive evidence has shown that human behavior is goal-based: goals guide perception, action selection [28], motivation [29] and evaluation of outcomes [30]. Goals of care are a central construct in clinical decision-making but may not be explicit in the EHR or may be buried in individual notes [31]. Patient-specific goals are often absent. Yet, both are crucial to organizing care [32,33]. Therefore, future EHRs will need to link clinicians’ goals of care with functional information as well as patient-specific goals. This linkage is key to making contextual information useful, especially in the elderly and for those with chronic conditions.

Temporality of contextual information is also a critical function for future EHRs. For example, changes in functional status are one of the best predictors of end of life [34]. However, this information is not currently stored as discrete, structured data. Clinicians’ abilities to identify changes, especially declines in function, depend upon their willingness to review the myriad clinical notes for relevant information. In the future, changes in function over time would be essential to represent in the medical record and would be especially important for care across the life continuum [35,36]. An alternative is to use patient- and caregiver-facing technologies to collect information on mental, social or physical status and integrate these data into the EHR. Linking the narrative to a structured data element during documentation would be ideal.

As mentioned earlier, the source of contextual information is important to clinicians. Sources have varying values, so knowing “who” and “how” the information was collected would add informational value. It may be possible to extract functional information from clinical notes using information extraction tools, such as NLP and present the information along with its source to support improved decision-making.

Designing EHRs to represent attributes of Information Quality would require noting the veracity of the data, which clinicians currently establish by looking for contradictions and redundancies in the record. An information display highlighting discrepancies in the data may be difficult to create, but would duplicate what clinicians report they do now as part of their decision-making.

In summary, in the next generation EHRs, contextual information should be linked to specific clinical and patient goals, include temporal attributes, and have identified source attribution. Several possible display formats may work, e.g., the use of trending, time stamps and progressive disclosure to identify source attributes. The most effective display may still need more research. Valdez and co-authors have suggested that displaying the patient’s context could best be done in terms of the “patient’s work”, a concept derived from the sociologist Strauss’ earlier research on the concept of “work” overall. This perspective allows the concepfct of agency and context to be more fully integrated into design [37].

In addition, psychological studies suggest that contextual information may be not be cognitively organized the same way as clinical information [38]. Clinical information can be represented numerically while social information is likely represented in “story-form’ and is personal, specific, and linked to a particular time and geography [39]. Sharing such contextual information might be better displayed using stories (narrative) and/or videos.

### Implications for team based care

Substantial research supports the link between teamwork and outcomes in a variety of settings and teams from intensive care settings, simulated events, surgical teams and operating rooms [40-43]. Across these studies, poor teamwork is correlated with negative outcomes while good teamwork results in fewer patient complications and better outcomes. A recent meta-analysis demonstrated the importance of emergent team cognition on team performance [44]. The degree to which team members share information, particularly information about who knows what (known as Transactive Memory), is critical to both team performance and team satisfaction [45]. Thus, a major component of contextual information display is to support team-based care and the creation of a shared mental model of the patient. One key component to successfully achieving this goal would be to integrate the various team member’s perspectives as well as to be able to integrate numerical assessments, pictures and narrative (a high bar!).

### Study limitations

This study has several limitations. First, it involves a single setting and one kind of health care delivery system. The sample is representative of the team composition for PCMH, and the setting is also reflective of current PCMH practices. The sample size is consistent with qualitative techniques. Secondly, the interviewers were clinicians, which might have added bias. This bias would be mitigated by the fact that all had advanced education in psychology or human factors.

Future studies could examine prototype EHR displays to improve contextual information displays and clinical decision-making. Researchers may explore the usefulness of narrative patient summaries versus more traditional information summaries in use today.

## Conclusions

This study identified clinician’s perceptions about important attributes of contextual information for clinical decision-making especially in computerized environments. Six themes about critical information attributes emerged during data analyses. Because contextual information is poorly represented in contemporary EHRs, finding the information, forming a coherent sense of the patient and integrating that information into the clinical care plans of the team is a significant challenge. However, improved EHR designs are needed to support coordinated patient care that meets the patient’s needs. Future work to improve cognitive support for contextually-based decisions will need to address the information dimensions identified here.
